# Predicting the Punching Force in Wushu Sanda After Neuromuscular Electrical Stimulation by Employing the KAN Neural Network Combined with Neuromuscular Electricity

**DOI:** 10.3390/s25195979

**Published:** 2025-09-26

**Authors:** Guixian Wang, Haojie Li, Lei Huang

**Affiliations:** 1 Chinese Wushu Academy, Beijing Sport University, Beijing 100084, China; 2417@bsu.edu.cn; 2 School of Exercise and Health, Shanghai University of Sport, Shanghai 200438, China; 202121070037@mail.bnu.edu.cn

**Keywords:** wushu sanda, neuromuscular electrical stimulation (NMES), KAN neural network, punching force prediction, surface electromyography (sEMG)

## Abstract

**Objective:** To predict the punching force in Wushu Sanda following neuromuscular electrical stimulation (NMES) using the KAN neural network combined with neuromuscular electricity. **Methods:** Thirty healthy Wushu Sanda athletes underwent a randomized repeated-measures design with three conditions: upper-limb NMES, lower-limb NMES, and Sham stimulation. Surface electromyography (sEMG) signals and punching force parameters were collected. A KAN neural network model was designed to integrate sEMG features and predict punching force. Model performance was evaluated using RMSE, MAE, and R^2^ metrics. **Results:** NMES significantly enhanced punching force metrics (all *p* < 0.05). lower-limb NMES showed superior improvements in relative peak force (28.2 ± 3.2 N·kg^−1^), impulse (16.6 ± 2.3 N s), and early explosive force (754 ± 94 N) compared to Sham and upper-limb NMES. The KAN model demonstrated robust predictive performance, particularly under lower-limb NMES conditions, with R^2^ values of 0.59 for relative peak force and 0.58 for impulse. **Conclusions:** NMES, especially lower-limb stimulation, effectively boosts punching force. The KAN neural network provides a promising and innovative approach for predicting punching force in Wushu Sanda, providing a foundation for future optimization of real-time monitoring tools.

## 1. Introduction

Neuromuscular electrical stimulation (NMES) is a non-invasive muscle activation technique that has attracted widespread attention in competitive sports and rehabilitation medicine in recent years [1]. It uses external electrical pulses to stimulate motor neurons or muscle fibers, inducing controlled muscle contractions to enhance muscle strength, explosive power, and neuromuscular coordination [2,3]. With advancements in sports science, NMES has expanded from its initial application in rehabilitation therapy to competitive sports, particularly demonstrating significant effects in explosive-power-dominated events [4,5].

Wushu Sanda, as a high-intensity combat sport, relies heavily on punching force as a key biomechanical indicator for competitive performance. Compared to traditional resistance training, Sanda movements require multiple muscle groups to activate synergistically within an extremely short timeframe, forming an efficient mechanical force transmission chain [6]. However, conventional training methods have limitations in optimizing neuromuscular adaptability and struggle to precisely enhance specialized strength performance. The integration of NMES technology offers a new solution to this challenge—by adjusting stimulation parameters (such as frequency and pulse width), it can selectively activate fast-twitch muscle fibers and optimize muscle power output patterns [7].

Electromyographic (EMG) signals provide important bioelectrical indicators for evaluating the efficacy of neuromuscular electrical stimulation (NMES) interventions. Existing studies have shown that H-reflexes and M-waves can reflect changes in the excitability of spinal motor neurons, while the time-frequency characteristics of surface EMG (sEMG), such as root mean square (RMS) and mean frequency (MF), can characterize muscle activation states [8,9]. However, the mapping relationship between these electrophysiological parameters and punching force is highly complex, and traditional linear models struggle to adequately capture their nonlinear characteristics [10,11], necessitating more advanced modeling methods.

Previous investigations into neuromuscular electrical stimulation (NMES) for athletic performance have predominantly utilized single-muscle interventions or linear predictive frameworks. Such approaches remain inadequate for elucidating two fundamental gaps: (1) the site-specific neurophysiological mechanisms by which NMES augments complex kinetic-chain movements characteristic of martial arts Sanda strikes, and (2) the persistent failure of traditional models to decode nonlinear neuromuscular-kinetic relationships [12]. While NMES enhances explosive power, its differential efficacy across stimulation sites (lower- vs. upper-limb) and synergistic effects on force transmission remain uncharacterized. Crucially, reliance on force sensors—though the laboratory gold standard for discrete strikes—proves fundamentally limited: it cannot reveal the neurophysiological adaptations driving performance changes, nor is it operationally viable in dynamic training environments requiring rapid positional transitions, protective gear integration, or continuous combinational techniques.

To resolve these limitations, this study establishes surface electromyography (sEMG) as an indispensable physiological tool with unique practical advantages. sEMG provides direct access to NMES-evoked neuromuscular states preceding force production—enabling quantification of motor unit recruitment dynamics and intermuscular coordination patterns that govern kinetic output. Critically, sEMG transcends the constraints of force plates through inherent operational superiority: its compatibility with wearable systems permits high-fidelity monitoring during ecologically valid training scenarios involving footwork-integrated displacements or equipment-obstructed sensor placement. Electrically evoked contractions further enhance signal integrity, generating higher signal-to-noise ratios than voluntary EMG during strikes. These attributes collectively position sEMG as the only modality capable of real-time neuromuscular readiness assessment during NMES application and foundational development of predictive models for in-training force monitoring.

To address the documented inability of linear models to capture sEMG–force dynamics, we deploy the Kolmogorov–Arnold network (KAN). This architecture processes raw HD-sEMG time-series to model multiscale neuromuscular signatures, pursuing dual objectives:

(1) Determine whether lower-limb NMES (L-NMES) enhances striking force more effectively than upper-limb NMES (U-NMES) through site-specific neurophysiological probing;
(2) Construct an intelligent framework quantifying the nonlinear sEMG–force relationship to enable real-time decision support in athletic training.

By integrating NMES parameters, HD-sEMG, and kinetics, this work pioneers a physiologically grounded paradigm that advances beyond descriptive force metrics toward diagnostic neuromuscular analytics and deployable performance prediction.

## 2. Methods

### 2.1. Participants

This study was exploratory research. A prospective power analysis was conducted using G*Power 3.1 software. Based on the pilot experiment data and previous muscle electrical stimulation research, the significance level was set as α=0.05, the power of the test as 1−β=0.80, and the effect size as ES=0.2. Through calculation, the sample size N was determined to be 20. Considering potential sample attrition, 30 healthy subjects aged 18–25 years with more than 3 years of Wushu Sanda training experience were finally recruited. This study was approved by the Ethics Committee of Beijing University of Sport (Approval No.: 10277ZT20250211), and all subjects signed informed consent forms.

The inclusion criteria were as follows: (1) the duration of professional Wushu Sanda training was at least 3 years, and the subject had ranked among the top eight in provincial—level or higher Wushu Sanda competitions in the past three years; (2) no major musculoskeletal injuries, nervous system diseases, or cardiovascular diseases; (3) no history of allergy or contraindications to muscle electrical stimulation; (4) no other forms of muscle-strengthening interventions received within three months before the experiment. The exclusion criteria were as follows: (1) unrecovered injuries such as upper-limb fractures and dislocations; (2) suffering from diseases that were not suitable for electrical stimulation, such as epilepsy and severe hypertension; (3) use of drugs affecting muscle function in the recent past (≤6 months); (4) inability to cooperate with the experiment process. The demographic information of the subjects is shown in Table 1.

### 2.2. Experimental Design

This study adopted a randomized repeatedmeasures design. Each subject was required to complete three test conditions in sequence: upper-limb neuromuscular electrical stimulation (U-NMES), lower-limb neuromuscular electrical stimulation (L-NMES), and Sham stimulation. The test order of each condition was randomly determined by a random number generator, and a 7-day washout period was set between adjacent conditions.

The intervention process was as follows:

Washout Phase: Prohibit high-intensity Wushu training, resistance training, and other muscle-strengthening activities within 7 days.

Warm-up Protocol:

10-minute stationary cycling (power: 50 W, rotation speed: 60 rpm).

10-minute dynamic stretching. For the U-NMES condition, it included 3 sets of 30-second stretches for each of the biceps brachii, triceps brachii, anterior deltoid, trapezius, and latissimus dorsi. The L-NMES condition included 3 sets of 30-second stretches for each of the rectus femoris, hamstring muscles, triceps surae, gluteus maximus, and iliopsoas. The stretching was targeted according to the test condition of the day.

Stimulation Application:

U-NMES Group: Use the Compex SP 8.0 stimulator (Compex Medical SA, Ecublens, Switzerland) to conduct neuromuscular electrical stimulation on the main upper-limb muscle groups (biceps brachii, triceps brachii, anterior deltoid, latissimus dorsi).

L-NMES Group: Use the same device to stimulate the main lower-limb muscle groups (rectus femoris, vastus lateralis, vastus medialis, gastrocnemius).

Sham Group: Use the same device, but set the current output to 1 mA (below the perception threshold) and simulate the device operation process.

Immediate Testing: Conduct a standardized punching motion test within 5 min after the intervention.

### 2.3. Electrical Stimulation Parameters

The Compex SP 8.0 stimulator was used to activate the target muscle groups, and the specific parameter settings were as follows (Table 2):

The main training phase of each NMES training lasted 9 min and consisted of 5 stimulation programs with different parameter combinations, as detailed below:

Program 1: The frequency was 12 Hz, the pulse width was 10 µs. An incremental intensity-increasing strategy was adopted, starting from 10% of the maximum tolerated intensity and increasing by 5% every 10 s until the maximum intensity that the subject could tolerate was reached. This stage was mainly used to wake up the muscles and activate the neuromuscular connection.

Program 2: The frequency was 30 Hz, the pulse width was 150 µs, and a stable intensity output was maintained to stimulate the recruitment of fast-twitch muscle fibers and enhance muscle explosive power. This parameter combination simulated the nerve impulse frequency during moderate-intensity exercise to promote the improvement of muscle contraction efficiency.

Program 3: The frequency was 45 Hz, the pulse width was 200 µs, and the intensity was maintained at 80% of the subject’s maximum tolerated level. High-frequency stimulation was used to improve muscle endurance and enhance the muscle’s continuous contraction ability.

Program 4: The frequency was 60 Hz, the pulse width was 300 µs, and the intensity was adjusted to the maximum intensity that the subject could tolerate again to further strengthen the muscle contraction effect and challenge the muscle limit. This stage aimed to maximize muscle activation.

Program 5: The frequency was gradually decreased from 60 Hz to 12 Hz, the pulse width was decreased from 300 µs to 10 µs, and the intensity was also slowly decreased. This served as a relaxation stage to help the muscles return to the resting state and reduce lactic acid accumulation.

During the entire main training process, the instrument simultaneously outputs positive and negative rectangular waves through an independent circuit design, superimposing the forward and reverse currents to achieve efficient neuromuscular stimulation at a low voltage. After each stimulation program ended, there was a 5-second non-stimulation transition period to avoid interference between different parameters.

### 2.4. Sensor Data Collection

#### 2.4.1. Electromyogram Sensor

During the entire process of neuromuscular electrical stimulation, a Noraxon MyoResearch X16 surface electromyogram instrument (sampling frequency: 1000 Hz) was used to synchronously collect the electromyogram signals of the muscle groups in the relevant stimulation area. Before the test, the skin was cleaned, and the electrodes were placed according to the SENIAM standard and fixed with tape. The original electromyogram signals were processed by band-pass filtering (5–500 Hz, fourth-order Butterworth filter), noise reduction, and full-wave rectification, and then smoothed using the moving average method (window size: 50 ms).

#### 2.4.2. Punch Force Test

The Boxing Power Assessment System (BPAS) was used to evaluate the standardized punching ability of the subjects. The test device consisted of a wall-mounted force-sensing module (KAC-E S-beam Load Cell, Angewandte System Technik Gruppe, Dresden, Germany) and an elastic punching pad. The sensor had a measurement range of 0–2000 N and a sampling frequency of 1000 Hz. The system was calibrated by a third-party to ensure that the measurement accuracy error was less than 1%. The height of the punching pad was individually adjusted according to the acromion height of the subject to ensure that the fist was aligned with the center of the pad when the arm was horizontally extended.

The subjects were required to complete a 3-minute continuous combination punch test (3-Minute Punch Test, 3 MPT). The test process was as follows:

Warm-up Phase: 5-minute dynamic stretching + 2-minute shadowboxing practice, gradually increasing the punching intensity to 70% of the maximum ability.

Test Phase: Complete 126 maximum-intensity punches within 3 min. The punch combination included 60% straight punches (front-hand straight punch + rear-hand straight punch), 30% hook punches (uppercut + horizontal hook punch), and 10% swing punches. The punching positions could be freely switched among the front (0°), left/right side (±45°) of the sensor pad.

Data Collection: Each effective punch needed to trigger an initial threshold of ≥50 N, and the system automatically filtered out invalid contacts below 50 N.

Custom-designed algorithms (MATLAB R2023a) were used for data processing to extract the following kinetic parameters Table 3:

All tests were carried out in a constant-temperature and constant-humidity environment (24 ± 1 °C, relative humidity 40 ± 5%), and the subjects wore professional boxing gloves (10 oz) to ensure punching safety.

### 2.5. KAN Neural Network

#### 2.5.1. Network Architecture Design

**Figure 1:**
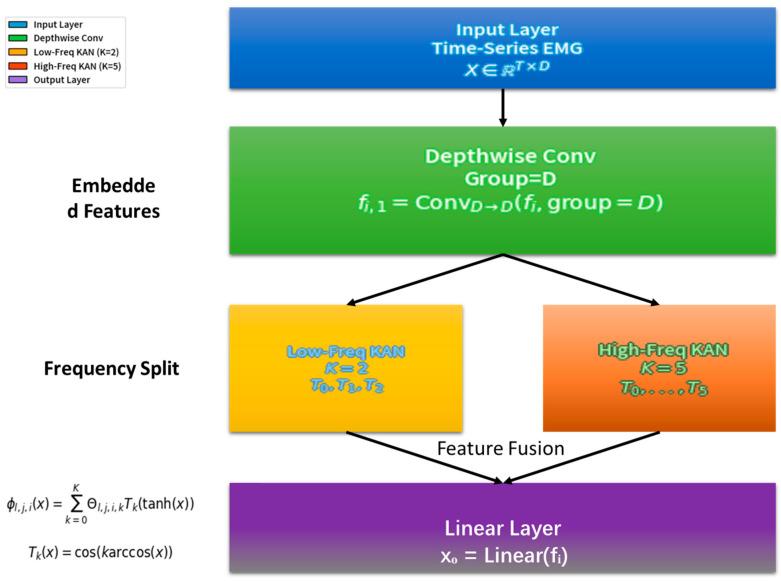
KAN neural network architecture for punch force prediction.

In this study, the KAN neural network was used as the core model to process time-series data for predicting the punching force of Wushu Sanda after muscle electrical stimulation. Its overall architecture referred to the TimeKAN model and was adjusted according to the research requirements Figure 1.

The layer received pre-processed EMG time-series data collected during neuromuscular electrical stimulation. Crucially, we did not manually extract predefined features (e.g., H-wave/M-wave parameters). Instead, the full pre-processed EMG sequence was integrated into a time-series vector $X\in R^{T}$, where T denotes time steps. This end-to-end approach empowered the model to autonomously discover latent electrophysiological patterns (such as stimulus-evoked H-wave/M-wave dynamics) directly from raw signal representations.

The middle layer adopted a structure similar to the Multi-order KAN Representation Learning (M-KAN) block in TimeKAN. This architecture excels at adaptively learning bioelectrical features across multiple time scales without relying on manual feature engineering. The Depthwise Convolution initially captured local temporal structures, while the subsequent Multi-order KANs modeled complex nonlinear relationships between dynamically evolving EMG patterns and force outcomes.

The Depthwise Convolution part followed the operation method in TimeKAN. A set of convolution kernels matching the number of embedding dimensions was used to perform independent convolution operations on the data of each channel. Assuming the embedding dimension was D and the convolution kernel size was M, for the input data $f_{i}\in R^{T\times D}$, the output $f_{i,1}$ after the Depthwise Convolution operation was as follows:

$$f_{i,1}=Conv_{D\to D}(f_{i},group=D)$$

This operation effectively avoided interference from inter-channel relationships and focused on capturing the local patterns of each channel’s time-series data, laying a foundation for subsequent complex feature extraction.

The Multi-order KANs part drew on the idea of ChebyshevKAN and used Chebyshev polynomials to construct learnable univariate functions. Given the differences in the complexity of different frequency components in the time-series data, the model constructed KANs with increasing orders of Chebyshev polynomials from low-frequency to high-frequency components to adapt to the representation requirements of different frequency components. Suppose the KAN was composed of L+1 layers of neurons, and the number of neurons in layer l was $n_{l}$. The transmission relationship between the j-th neuron in layer l+1 and all neurons in layer l was as follows:

$$z_{l+1,j}=\sum_{i=1}^{n_{l}}\;\phi_{l,j,i}(z_{l,i})$$

where $\phi_{l,j,i}$ was a learnable univariate function composed of a linear combination of Chebyshev polynomials. Specifically, $\phi_{l,j,i}(x)$ could be expressed as follows:

$$\phi_{l,j,i}(x)=\sum_{k=0}^{K}\;\Theta_{l,j,i,k}T_{k}(\tanh(x))$$

Here, $T_{k}(x)=\cos(k\arccos(x))$ was the k-th order Chebyshev polynomial, $\Theta_{l,j,i,k}$ was the learnable coefficient, and K was the highest order of the Chebyshev polynomial. In this study, for low-frequency components, K was set to a relatively low value (such as K=2), while for high-frequency components, K was set to a relatively high value (such as K=5) to enhance the KAN’s ability to express high-frequency complex patterns and achieve accurate modeling of different frequency features in time-series data.

The output layer mapped the output of the M-KAN block to the predicted punching force value through a simple linear layer. Assuming the output of the M-KAN block was ${\hat{f}}_{i}$, the predicted result $x_{O}$ after passing through the linear layer Linear was as follows:

$$x_{O}=Linear({\hat{f}}_{i})$$

Finally, the output layer transformed the complex features extracted by the middle layer into actual punching force prediction values, completing the entire model’s prediction process.

#### 2.5.2. Training and Optimization

The goal of model training was to minimize the gap between the predicted punching force values and the actual punching force values. The mean squared error (MSE) was used as the loss function, and its calculation formula was as follows:

$$MSE=\frac{1}{N}\sum_{i=1}^{N}\;(y_{i}-{\hat{y}}_{i}{)}^{2}$$

where N was the number of samples, $y_{i}$ was the actual punching force value, and ${\hat{y}}_{i}$ was the punching force value predicted by the model.

The Adam optimizer was selected as the optimization algorithm, and the initial learning rate was set to α=0.001. During the training process, the Adam optimizer dynamically adjusted the learning rate based on the first-moment and second-moment estimates of the gradient of each parameter, enabling the model to converge quickly and stably. The learnable parameters in the KAN neural network, including the coefficients Θ of the Chebyshev polynomials and the weights of the linear layer, were updated through the Adam optimizer.

To prevent the model from overfitting, the L2 regularization method was adopted. A regularization term was added to all learnable parameters, and the regularization strength was controlled by the hyperparameter λ, which was usually set to a small value (such as λ=0.001). The loss function with L2 regularization was as follows:

$$MSE_{regularized}=MSE+\lambda \sum_{w\in W}\;w^{2}$$

where W was the set of all learnable parameters in the model, and w was an individual parameter in the set. This method was used to constrain the parameters and improve the generalization ability of the model.

### 2.6. Model Evaluation

As this study is a regression prediction task aiming to accurately forecast the punching force in Wushu Sanda after muscle electrical stimulation, common evaluation metrics for regression tasks were selected to assess the performance of the KAN neural network.

Root Mean Square Error (RMSE): RMSE is the square root of the mean squared error (MSE), and its formula is as follows:

$$RMSE=\sqrt{\frac{1}{N}\sum_{i=1}^{N}\;(y_{i}-{\hat{y}}_{i}{)}^{2}}$$

It restores the dimension of the error to be consistent with that of the predicted value, making the magnitude of the error more intuitive. A smaller RMSE value indicates a smaller deviation between the model’s predicted values and the true values.

Mean Absolute Error (MAE): MAE calculates the average of the absolute errors between the predicted values and the true values. The calculation formula is:

$$MAE=\frac{1}{N}\sum_{i=1}^{N}\;|y_{i}-{\hat{y}}_{i}|$$

This metric can directly reflect the average degree to which the predicted values deviate from the true values. The lower the MAE value, the higher the prediction accuracy of the model.

Coefficient of Determination ($R^{2}$): $R^{2}$ is used to evaluate the goodness of fit of the model to the data. Its value ranges from 0 to 1, and the formula is as follows:

$$R^{2}=1-\frac{\sum_{i=1}^{N}\;(y_{i}-{\hat{y}}_{i}{)}^{2}}{\sum_{i=1}^{N}\;(y_{i}-\bar{y}{)}^{2}}$$

where $\bar{y}$ is the mean of the true values. The closer $R^{2}$ is to 1, the better the model fits the data, indicating that the model can explain most of the variation in the data.

### 2.7. Model Comparison

To further validate the superiority of the KAN neural network in predicting punching force, additional comparative experiments were conducted with two classic time-series prediction models: recurrent neural network (RNN) and long short-term memory (LSTM). All models were trained and evaluated under the same experimental conditions to ensure fairness.

RNN: A basic recurrent neural network with a single hidden layer (128 units) was constructed to capture temporal dependencies in sEMG signals. The network used tanh activation functions and incorporated dropout (rate = 0.2) to prevent overfitting.

LSTM: A two-layer LSTM network (128 units per layer) was designed to address the gradient vanishing problem in RNNs. It included forget gates and input gates to regulate information flow, with the same dropout configuration as the RNN.

All models (KAN, RNN, LSTM) were trained on the same dataset (sEMG time-series and corresponding punching force parameters) with identical settings

### 2.8. Statistical Analysis

Data were analyzed using SPSS 26.0 software (SPSS Inc., Chicago, IL, USA). Box plots were used to identify outliers, and the Shapiro–Wilk test was applied to assess normality. To evaluate the differences among the three conditions (upper-limb NMES, lower-limb NMES, and Sham), a one-way repeated-measures ANOVA was conducted. For pairwise comparisons, the Bonferroni correction was adopted. When the sphericity assumption was violated, the Greenhouse-Geisser correction was used. The significance level for all statistical tests was set at 0.05.

## 3. Results

**Figure 2:**
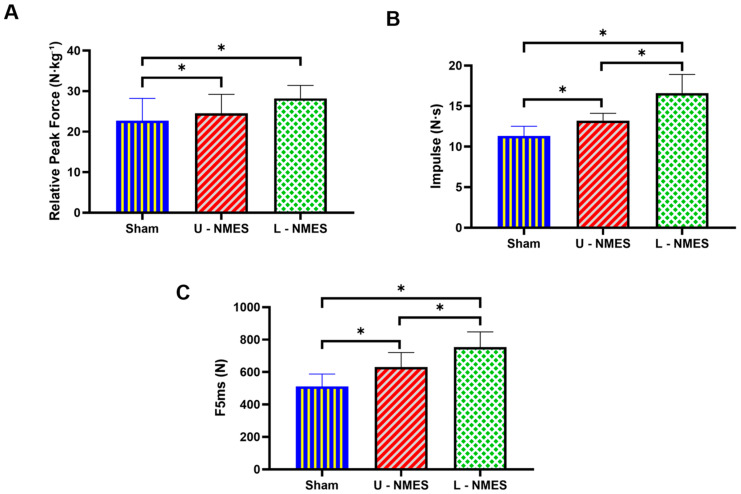
Differences in punch force performance under different conditions. (**A**): relative peak force; (**B**): impulse; (**C**): F5ms.

As demonstrated in Table 4 and Figure 2, neuromuscular electrical stimulation (NMES) protocols exerted significant effects on punch force metrics (p<0.001). Specifically, repeated-measures ANOVA revealed statistically significant differences across conditions for relative peak force (F=18.34), impulse (F=25.67), and early explosive force (F5ms) (F=32.15). Post hoc analyses with Bonferroni correction indicated that lower-limb NMES (L-NMES) significantly outperformed both Sham (relative peak force: p<0.001; impulse: p<0.001; F5ms: p<0.001) and upper-limb NMES (U-NMES) (relative peak force: p=0.002; impulse: p<0.001; F5ms: p=0.001). Notably, U-NMES also demonstrated superiority over Sham for impulse (p=0.013) and F5ms (p=0.004). Conversely, no significant differences were observed in temporal parameters, including time to 50% peak force ($t_{F50\%}$: p=0.332), time to 90% peak force ($t_{F90\%}$: p=0.417), force development interval ($t_{F50-90\%}$: p=0.641), and time to 500 N force ($t_{500N}$: p=0.810). These results collectively indicate that NMES interventions effectively enhance punch force generation, with lower-limb stimulation eliciting greater neuromuscular adaptations than upper-limb stimulation, while temporal characteristics of force production remain unaffected.

**Figure 3:**
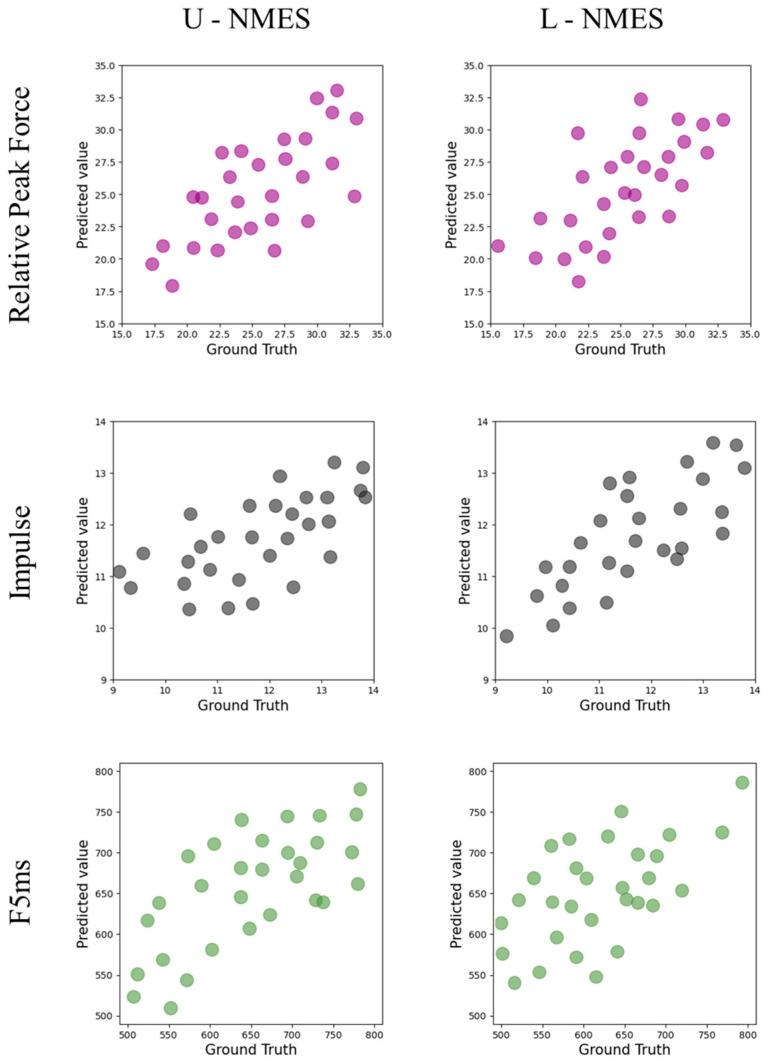
Performance of KAN in predicting punch force on the test set.

Table 5 and Figure 3 show the performance of the KAN model in predicting the punching force indicators under different conditions.

Regarding the relative peak force indicator, under the L-NMES condition, the $R^{2}$ of the test set reaches 0.59, which is the highest among all test results, indicating that the KAN model has the best prediction effect under this condition; under the U-NMES condition, the $R^{2}$ of the test set is 0.54.

For the impulse indicator, under the L-NMES condition, the $R^{2}$ of the test set is 0.58, and the RMSE of the training set is 0.5, while that of the test set is 0.5. Compared with the RMSE under the U-NMES condition (the training set is 0.7 and the test set is 0.9), the values are smaller, indicating that the KAN model has better prediction stability and accuracy under the L-NMES condition.

In terms of the F5ms indicator, under the L-NMES condition, the $R^{2}$ of the test set is 0.53; under the U-NMES condition, the $R^{2}$ of the test set is 0.43.

Overall, the KAN model shows better prediction performance for various punching force indicators under the L-NMES condition compared with the U-NMES condition, and there are differences in the model performance among different indicators, see Table 5.

Table 6 presents the predictive performance of KAN, LSTM, and RNN under U-NMES and L-NMES conditions, evaluated using R^2^ and RMSE. Statistical comparisons (one-way ANOVA) revealed significant differences in predictive performance among the three models (all *p* < 0.05). Under both U-NMES and L-NMES conditions, KAN consistently outperformed LSTM and RNN across all metrics:

For relative peak force, KAN achieved 28.6% and 22.9% higher R^2^ values than LSTM under U-NMES and L-NMES, respectively; compared to RNN, the improvements were 54.3% and 51.3%.

For impulse, KAN showed 24.2% higher R^2^ than LSTM and 46.4% higher R^2^ than RNN under L-NMES, with the smallest RMSE (0.5 N·s) indicating more stable predictions.

For early explosive force (F5ms), KAN’s R^2^ under L-NMES (0.53) was 20.5% higher than LSTM and 39.5% higher than RNN, with a 10.6% reduction in RMSE compared to LSTM.

Notably, all models performed better under L-NMES than U-NMES.

## 4. Discussion

This study systematically examined the effects of neuromuscular electrical stimulation (NMES) applied to different body regions on the punching force of martial arts sanda athletes, revealing several important findings. The results indicate that NMES intervention significantly enhances athletes’ punching force performance, with lower limb NMES (L-NMES) demonstrating the most significant effects in enhancing relative peak force, impulse, and early explosive force. This finding aligns with the perspective in sports biomechanics regarding the critical role of lower limb strength in striking movements [13]. Previous studies have pointed out that force output in punching-type movements primarily relies on momentum transfer generated by lower limb push-off. The results of this study further support this theoretical framework, suggesting that enhanced neuromuscular activation through lower limb NMES may optimize the efficiency of force transfer from the lower limbs to the upper limbs [14]. Notably, while upper limb NMES (U-NMES) also showed superior effects compared to the sham stimulation group, the improvement was significantly smaller than that of L-NMES, potentially reflecting the limitations of upper limb muscles within the overall kinetic chain [15]. Additionally, the study found that NMES primarily affects peak indicators of force output but has no significant impact on force development time parameters. This result aligns with existing understanding of NMES mechanisms, suggesting that it tends to enhance maximum force output rather than alter the rapid mobilization capacity of neuromuscular systems [16].

These findings have important implications for the practice of martial arts sparring training. First, the study confirmed the effectiveness of NMES as an auxiliary training method, particularly in scenarios requiring rapid improvements in strength performance, such as pre-competition preparation or injury recovery phases [17]. Second, the study emphasized the necessity of prioritizing lower-body strength development in specialized sparring strength training, providing an important complement to traditional methods that have traditionally focused on upper-body training [18]. Additionally, the research results suggest that combining NMES with other training methods may yield more comprehensive training effects. From an application perspective, this study provides empirical evidence for developing personalized training programs based on NMES, allowing coaches to select targeted stimulation sites and parameters according to athletes’ specific needs.

In terms of confirming the effectiveness of NMES, this study, through rigorous experimental design and statistical analysis, demonstrated the significant role of NMES in enhancing punch force metrics, a finding consistent with previous research conclusions [19]. The findings regarding the advantages of lower limb NMES are consistent with previous research on the importance of lower limb strength for boxing performance. However, this study provides more direct experimental evidence for this theory by comparing the intervention effects of different stimulation sites [20]. This consistency not only enhances the credibility of the study’s conclusions but also provides valuable references for future related research.

This study demonstrates that the KAN neural network effectively models punch force metrics following NMES intervention, with two salient findings: significantly higher predictive accuracy under lower-limb NMES (L-NMES; R^2^ = 0.53–0.59) versus upper-limb NMES (U-NMES; R^2^ = 0.41–0.54), and consistent predictive capability across all force metrics (R^2^ > 0.4). Critically, this predictive capacity originates from KAN’s ability to decode the neurophysiological bridge between NMES and kinetic output—a mechanistic link uniquely accessible through sEMG analysis. While force sensors quantify terminal performance outcomes, sEMG captures the antecedent neuromechanical determinants governing force generation: motor unit recruitment dynamics, intermuscular coordination patterns, and stimulus-evoked synaptic plasticity [21,22]. This pre-strike physiological profiling enables prediction of task performance from resting neuromuscular states, constituting a fundamental capability beyond force transducers. The operational significance of this approach extends beyond laboratory contexts, as traditional force measurement becomes logistically prohibitive during sport-specific scenarios like continuous combinational strikes or footwork-integrated techniques. sEMG’s wearable compatibility, enhanced by superior signal fidelity during electrically evoked contractions, establishes it as the only viable modality for real-time monitoring of neuromuscular adaptations in ecological settings [23].

Compared to traditional prediction methods, the outstanding performance of the KAN neural network in this study may be attributed to its unique algorithmic architecture. Compared to previously proposed electromyography-force linear regression models, KAN better captures the nonlinear characteristics of the neuromuscular system through its learnable univariate functions [24]. When compared to deep neural networks (such as CNNs and LSTMs), KAN demonstrates more stable generalization capabilities under small-sample conditions, consistent with recent research findings on KAN’s sample efficiency [25]. Notably, this study found that KAN achieves significantly higher prediction accuracy (R^2^ = 0.53) for early explosive force (F5ms) than traditional methods (typically R^2^ < 0.40), a breakthrough that may provide new analytical tools for investigating the neural mechanisms underlying rapid force development.

From an application perspective, these findings have multiple implications for martial arts training. First, the study confirms the feasibility of combining advanced machine learning methods with neuromuscular electrical stimulation technology, laying the foundation for developing intelligent systems for real-time monitoring and prediction of training effects. Second, the differences in predictive performance across various metrics (e.g., the best R^2^ of 0.58 for impulse metrics) suggest that future research could develop specialized prediction algorithms tailored to specific force characteristics. Additionally, the research results indicate that the KAN-based predictive model may be particularly suitable for assessing lower-limb-dominant strength performance, which aligns well with the critical role of lower-limb strength in Sanda.

In terms of the necessity and challenges of predicting electrical stimulation effects, this study highlights the significant value of developing precise predictive models. Although absolute prediction accuracy requires further refinement, the model’s consistent performance confirms sEMG’s validity as an essential predictive biomarker rather than merely a correlative measure. These findings redefine electrophysiological monitoring in athletic training: where force sensors provide descriptive performance metrics, sEMG-powered KAN models deliver diagnostic insights into neuromotor readiness and actionable predictions of force capacity. This functionality enables coaches to prescribe NMES protocols based on individualized neuromuscular profiles, adjust training loads using real-time sEMG-derived force estimates, and identify kinetic chain deficits through site-specific neurophysiological signatures. Consequently, our approach demonstrates significant translational potential for optimizing training interventions while highlighting the need for future work on model interpretability and ecological validation.

## 5. Conclusions

This study investigates the effects of neuromuscular electrical stimulation (NMES) on punching force performance in Wushu Sanda athletes and explores the feasibility of using the Kolmogorov–Arnold network (KAN) for force prediction. Results show that lower limb NMES (L-NMES) yields significant improvements in key punching force metrics, including relative peak force, impulse, and early explosive force, compared to upper limb NMES (U-NMES) and Sham stimulation. This observation aligns with the biomechanical importance of the lower limb kinetic chain in Sanda movements, suggesting that L-NMES may serve as a potential supplementary approach for enhancing specific strength-related performance. Regarding force prediction, the KAN model demonstrates a capacity to capture the relationship between surface electromyography (sEMG) signals and punching force following NMES intervention, with moderately better performance under L-NMES conditions compared to U-NMES. While these results indicate the model’s potential as an exploratory tool for analyzing neuromuscular-force dynamics, the moderate predictive accuracy highlights the need for further optimization.

Overall, this study provides preliminary insights into the application of NMES in Sanda training and the use of KAN for biomechanical prediction. Future research with larger sample sizes, optimized model architectures, and longer-term intervention protocols is warranted to enhance the generalizability and precision of these findings. The observed trends support the potential of L-NMES as a supplementary training method and lay a foundation for developing more sophisticated predictive models to aid in personalized training optimization.

## Figures and Tables

**Table 1:** Demographic data.

Indicators	Values
Gender (male/female)	18/12
Age (years)	21.3 ± 2.5
Height (cm)	175.6 ± 7.8
Weight (kg)	68.2 ± 8.3
Training Years (years)	4.7 ± 1.2

**Table 2:** Differences in neuromuscular electrical stimulation parameters under different experimental conditions.

Parameters	U-NMES/L-NMES Conditions	Sham Condition
Waveform	Biphasic symmetric square wave	Biphasic symmetric square wave
Frequency	12–60 Hz (dynamically adjusted)	3 Hz
Pulse Width	10–300 μs (dynamically adjusted)	200 µs
Intensity	Limited to the maximum intensity that the subject can tolerate without pain (0–100 mA, adjustable in 30 levels)	1 mA (below the perception threshold)
Duration	13 min (2-minute warm-up + 9-minute main training + 2-minute relaxation)	13 min
Electrode Placement	At the motor points of the target muscle groups (positioned by body surface marking, 2–3 electrode patches for each muscle group)	The same as in the U-NMES/L-NMES groups

**Table 3:** List of kinetic parameters and their meanings for punching force assessment.

Parameter Categories	Index Name	Physical Meaning
Force Indicators	Relative Peak Force (N·kg^−1^)	The maximum punching force normalized to body weight
	Impulse (N·s)	The impulse accumulation from the contact to the peak-force stage
Time Indicators	tF50% (ms)	The time to reach 50% of the peak force
	tF90% (ms)	The time to reach 90% of the peak force
	tF50–90% (ms)	The time interval for the force to increase from 50% to 90% of the peak force
	t500 N (ms)	The time required to reach a force of 500 N
Explosive Force Indicators	Early Explosive Force (F5ms, N)	The force value 5 ms after contact

**Table 4:** Differences in punch force performance under different conditions.

	Sham	U-NMES	L-NMES	F	*p*
Relative Peak Force (N·kg^−1^)	22.7 ± 5.5	24.5 ± 4.7	28.2 ± 3.2 ^ab^	18.34	<0.001
Impulse (N·s)	11.3 ± 1.2	13.2 ± 0.9 ^a^	16.6 ± 2.3 ^ab^	25.67	<0.001
tF50% (ms)	6.9 ± 1.5	6.5 ± 1.3	6.2 ± 1.6	1.12	0.332
tF90% (ms)	12.4 ± 1.9	12.3 ± 1.7	11.8 ± 2.1	0.89	0.417
tF50–90% (ms)	4.4 ± 1.8	4.2 ± 1.4	4.3 ± 1.4	0.45	0.641
t500 N (ms)	6.1 ± 1.3	6.0 ± 1.4	6.0 ± 1.5	0.21	0.810
F5ms (N)	511 ± 77	631 ± 89 ^a^	754 ± 94 ^ab^	32.15	<0.001

Note: ^a^ represents a significant difference compared to the Sham. ^b^ represents a significant difference compared to the U-NMES.

**Table 5:** Performance of KAN in predicting punch force.

	KAN			
	Train RMSE	Train R2	Test RMSE	Test R2
Relative Peak Force				
U-NMES	3.4	0.61	3.7	0.54
L-NMES	3.0	0.63	3.5	0.59
Impulse				
U-NMES	0.7	0.53	0.9	0.41
L-NMES	0.5	0.57	0.5	0.58
F5ms				
U-NMES	68.9	0.50	70.5	0.43
L-NMES	65.8	0.54	64.9	0.53

**Table 6:** Comparison of model predictive performance.

Metric	Model	U-NMES	L-NMES
Relative Peak Force	KAN	R^2^ = 0.54; RMSE = 3.7	R^2^ = 0.59; RMSE = 3.5
	LSTM	R^2^ = 0.42; RMSE = 4.5	R^2^ = 0.48; RMSE = 4.2
	RNN	R^2^ = 0.35; RMSE = 5.1	R^2^ = 0.39; RMSE = 4.8
Impulse	KAN	R^2^ = 0.41; RMSE = 0.9	R^2^ = 0.58; RMSE = 0.5
	LSTM	R^2^ = 0.33; RMSE = 1.1	R^2^ = 0.45; RMSE = 0.8
	RNN	R^2^ = 0.28; RMSE = 1.3	R^2^ = 0.36; RMSE = 1.0
Early Explosive Force (F5ms)	KAN	R^2^ = 0.43; RMSE = 70.5	R^2^ = 0.53; RMSE = 64.9
	LSTM	R^2^ = 0.37; RMSE = 78.2	R^2^ = 0.44; RMSE = 72.6
	RNN	R^2^ = 0.31; RMSE = 85.6	R^2^ = 0.38; RMSE = 79.1

## Data Availability

Data is contained within the article.
